# The Application of the Open Pharmacological Concepts Triple Store (Open PHACTS) to Support Drug Discovery Research

**DOI:** 10.1371/journal.pone.0115460

**Published:** 2014-12-18

**Authors:** Joseline Ratnam, Barbara Zdrazil, Daniela Digles, Emiliano Cuadrado-Rodriguez, Jean-Marc Neefs, Hannah Tipney, Ronald Siebes, Andra Waagmeester, Glyn Bradley, Chau Han Chau, Lars Richter, Jose Brea, Chris T. Evelo, Edgar Jacoby, Stefan Senger, Maria Isabel Loza, Gerhard F. Ecker, Christine Chichester

**Affiliations:** 1 Universidade de Santiago de Compostela, Grupo BioFarma-USEF, Departamento de Farmacología, Campus Universitario Sur s/n, 15782 Santiago de Compostela, Spain; 2 University of Vienna, Department of Pharmaceutical Chemistry, Althanstrasse 14, 1090 Vienna, Austria; 3 Janssen Research & Development, Turnhoutseweg 30, Beerse, Belgium; 4 Swiss Institute of Bioinformatics, CALIPHO Group, CMU – Rue Michel-Servet 1, 1211 Geneva 4, Switzerland; 5 GSK Medicines Research Centre, Gunnels Wood Road, Stevenage, Hertfordshire, SG1 2NY, United Kingdom; 6 Vrije Universiteit, Faculty of Sciences, division of Math. and Computer Science, De Boelelaan 1081a, 1081 HV Amsterdam, The Netherlands; 7 Department of Bioinformatics – BiGCaT, Maastricht University, Maastricht, The Netherlands; Koc University, Turkey

## Abstract

Integration of open access, curated, high-quality information from multiple disciplines in the Life and Biomedical Sciences provides a holistic understanding of the domain. Additionally, the effective linking of diverse data sources can unearth hidden relationships and guide potential research strategies. However, given the lack of consistency between descriptors and identifiers used in different resources and the absence of a simple mechanism to link them, gathering and combining relevant, comprehensive information from diverse databases remains a challenge. The Open Pharmacological Concepts Triple Store (Open PHACTS) is an Innovative Medicines Initiative project that uses semantic web technology approaches to enable scientists to easily access and process data from multiple sources to solve real-world drug discovery problems. The project draws together sources of publicly-available pharmacological, physicochemical and biomolecular data, represents it in a stable infrastructure and provides well-defined information exploration and retrieval methods. Here, we highlight the utility of this platform in conjunction with workflow tools to solve pharmacological research questions that require interoperability between target, compound, and pathway data. Use cases presented herein cover 1) the comprehensive identification of chemical matter for a dopamine receptor drug discovery program 2) the identification of compounds active against all targets in the Epidermal growth factor receptor (ErbB) signaling pathway that have a relevance to disease and 3) the evaluation of established targets in the Vitamin D metabolism pathway to aid novel Vitamin D analogue design. The example workflows presented illustrate how the Open PHACTS Discovery Platform can be used to exploit existing knowledge and generate new hypotheses in the process of drug discovery.

## Introduction

While the approval rates for new drugs may be somewhat stable, pharmacological data of increasing size, dimensionality and complexity is being housed in public and proprietary databases [1], [2]. Within these separate data pools resides valuable scientific information that can help in the design of novel drugs, for example by predicting protein interactions with novel compounds [3], [4], [5], suggesting novel molecules with better properties or by finding existing chemical matter to test against a newly identified target. However, gathering relevant and comprehensive information from diverse sources is complicated; differences in data formats, the need for separate interfaces and query mechanisms, the lack of consistency between descriptors and identifiers in different resources and the absence of a simple mechanism to link them make this task non-trivial [6], [7]. Manual searches across different databases are tedious and time consuming, and thus often limited to individual compounds or targets only. The manual collation of data can be error prone and incomplete, of variable quality, and may not routinely capture the provenance of the original data sources. Moreover, for the effective and systematic combination and integration of complex data, the scientist analyst is required to possess an in-depth knowledge of the data models and licensing for each of a large set of systems. In addition, the need for bio- and chemo-informatics expertise and the ability to post-process any data retrieved makes this approach less accessible for a large majority of users. It is clear that many members of the drug discovery community will benefit greatly from accessible and well-structured data combined with useful analytics. For example, an integrated and comprehensive interface to publicly available pharmacology, physicochemical and biomolecular data could support initial drug screening stages and limit expensive late-stage trial failure. Such tools would also be invaluable to academia and small to medium enterprises (SMEs), which have historically enjoyed little access to proprietary integrated platforms.

A recent approach to address these issues is the integration of data from different sources by means of semantic web technologies [8], [9], [10]. The Open Pharmacological Concepts Triple Store (Open PHACTS) is an Innovative Medicines Initiative Knowledge Management project (IMI - 2nd call 2009) focusing on the application of semantic web technologies to overcome data access and knowledge integration challenges which can hinder current drug discovery efforts. The Open PHACTS Discovery Platform offers solutions for access to multiple, disparate and heterogeneous information sources, lack of standards and common identifiers for domain entities, and provides a means to interrogate the system with complex research questions [6], [7]. By drawing together multiple sources of publicly-available biomolecular, pharmacological and physicochemical data, Open PHACTS offers a state of the art platform that responds to structured, well defined queries in a meaningful and reproducible way (see S1 Table for currently available resources). An important functionality to maximise usefulness, especially in the pharmaceutical industry, is the ability to offer secure access to the Open PHACTS Discovery Platform. Presently, a robust security policy has been developed with a commercial triple store provider, Open Link (an Open PHACTS consortium partner), to supply the requisite privacy mechanisms.

As a collaboration between multiple European universities, the European Federation of Pharmaceutical Industries and Associations (EFPIA), and various SMEs (http://www.openphacts.org/partners/consortium), the Open PHACTS project benefits from a wealth of market experience and technical expertise. Development of the Open PHACTS Discovery Platform is driven in an agile, stepwise fashion focused on scientific competency questions and use cases for analysis of underlying data concepts and associations [11]. This approach ensures delivery of a platform ready and able to support drug discovery and development in both the public and private sector. A drug discovery focused Open PHACTS ‘Researchathon’ event (attended by 18 scientists from 8 academic institutions and 2 EFPIA companies) in 2013 identified critical requirements in terms of the specific datasets, functionalities and Application Programming Interface (API) calls which have shaped the Open PHACTS Discovery Platform development necessary to answer the specific questions presented here. The complete list of participants can be found here: http://www.openphacts.org/documents/events/130424_Researchathon_London_Participant%20List.pdf.

The aim of the present work is to highlight how the Open PHACTS Discovery Platform has been used by academic and pharmaceutical industry drug discovery scientists for the integration of public and proprietary pharmacology resources to i) identify target-specific chemical compounds, ii) support pathway-driven drug discovery. We describe how the platform can be used to solve common queries that require linkage of the entities of targets, compounds, and pathways, using the examples of a single target, Dopamine Receptor D_2_, and two well curated pathways of therapeutic interest from the public resource WikiPathways [12], ErbB signaling and Vitamin D metabolism (for detailed pathway selection criteria see S1 Method). As the platform is designed to be easily accessible from computational workflow systems, we show how the modularization of tasks using the Open PHACTS API [7] as well as full integration with pipelining tools can create workflows to answer complex queries around the selected examples. The workflow tools used herein are KNIME [13], a widely used, open-source graphical workbench to create and run workflows between executable ‘nodes’ and Pipeline Pilot [14], a proprietary workflow tool built on the Accelrys Enterprise Platform that similarly uses configurable ‘components’ to automate the process of accessing, analyzing and reporting scientific data.

Here, we demonstrate the utility of Open PHACTS in early drug discovery projectsthrough the development and application of workflows based on the Open PHACTS API and pipelining software, thereby allowing scientists to find answers to complex research questions requiring a wide range of data sources.

## Methods

### Open PHACTS API, databases, and workflow tools

All use case workflows utilized the Open PHACTS API version 1.3 (https://dev.openphacts.org/docs/1.3. Accessed 2014 Nov 30) to query across integrated public data sources: ChEMBL [15], [16], ChEBI [17], [18], [19], Drugbank [20], Chemspider [21], Gene Ontology (GO) [22], [23], WikiPathways [12], Uniprot [24], ENZYME [25] and ConceptWiki [26] (S1 Table). These data are available for download from the different data providers, under licensing models, such as Creative Commons Attribution (CC-BY), which require mostly citation and attribution for their reuse. The Open PHACTS consortium has endeavored to clarify and align data and software licenses to remove any barriers to use. The current resources, discussion of issues, and help documents are available on the Open PHACTS support site: http://support.openphacts.org/support/home (Accessed 2014 Nov 30). Proprietary databases used in Use Case A are: GVKBio GOSTAR (www.gostardb.com), Thomson Reuters (integrity.thomson-pharma.com) and in-house pharmacology databases from Janssen.

Use case workflows were constructed in the following manner: 1) entities of interest (targets, compounds, pathways, bioactivities, etc.) needed for the specific step in the workflow were identified, 2) URIs for the entities of interest were determined, 3) Open PHACTS API calls were executed, 4) results were parsed, 5) the steps were repeated multiple times if answers to previous cycles were needed to reach the final question. For each use case, the tasks were automated using the two most common cheminformatics workflow tools, namely Pipeline Pilot (http://accelrys.com/products/pipeline-pilot/) and KNIME version 2.9 (http://www.knime.com).

A custom Pipeline Pilot component library was co-developed with Accelrys to access the Open PHACTS API calls and parse the output. These components were used for the Use Case A workflow and are available on the Open PHACTS page on the Accelrys community website at (https://community.accelrys.com/docs/DOC-6473. Accessed 2014 Nov 30).

A series of generic KNIME utility nodes (https://github.com/openphacts/OPS-Knime. Accessed 2014 Nov 30) were created to incorporate the Open PHACTS services into the KNIME workbench. These nodes use two-dimensional tables, such as named rows and columns, as input and generate equivalent output. Since the Open PHACTS API services produce nested output (e.g. JSON or XML), a KNIME 'unfolding' algorithm was implemented as a node, transforming the Open PHACTS output into a KNIME table. The Open PHACTS API services are described in the Swagger REST service description format, enabling automatic generation of templates in KNIME. The result of running this utility node is a URL that represents the desired service call within a workflow. These nodes were used to construct workflows for Use Cases B and C.

**Figure 1:**
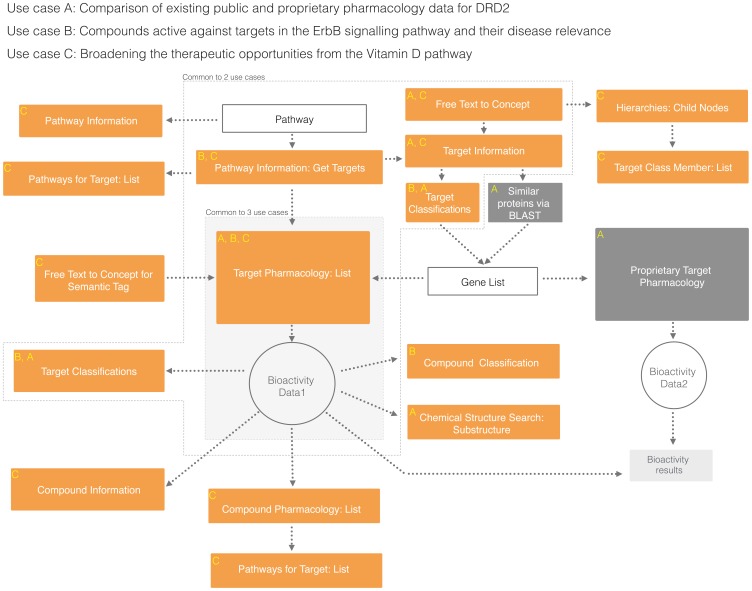
Open PHACTS v1.3 API calls (orange boxes) used to address use cases A, B and C, as described in Methods. Operations performed outside Open PHACTS, viz., sequence similarity searches via BLAST and access to proprietary databases (dark grey boxes) are facilitated by information derived from the platform. Sample input URIs for each API call is shown in S2 Table.

An overview of the API calls used to construct workflows for all use cases is represented in Fig. 1.

### Internal dictionaries for standardizing target, compound, and bioactivity nomenclature in proprietary databases

Use Case A required prior resolution of non-standard identifiers for compounds, targets and bioactivities present in proprietary pharmacology databases. As such, tautomeric SMILES nomenclature was selected for compounds, human gene symbols for targets, and log-transformation for bioactivity data, as these standards are stable and offer possibilities for integration with additional data types. To align external databases with EFPIA in-house data that traditionally use legacy gene symbols and not community accepted standard identifiers, a mapping table was created to link pharmacology database fields with HUGO gene symbols. An internal dictionary was created for each database to map the drug target keywords to HUGO gene symbols, and this information was added back to target information when necessary.

We also ensured that results from Open PHACTS would map to the different database fields by strictly adhering to target dictionaries and field mappings in a Pipeline Pilot protocol.

### Generating a list of related targets (gene names)

In order to expand pharmacology data to related proteins, three strategies are possible: finding targets linked to the same GO concept in Open PHACTS (the ‘Target Classifications’ API call), using the target protein sequence in a BLAST [27] alignment to obtain UniProt identifiers of related proteins (by sequence), or by manual collection of protein identifiers from literature or protein family databases. In all cases, Open PHACTS can be used to obtain gene names correlated with UniProt identifiers The related proteins retrieved from these methods may represent splice variants, orthologues or homologous paralogues. In the following use cases the distinction between these cases were not investigate, although they could potentially have some influence on the number of pharmacological records retrieved from Open PHACTS. In the case of a well-studied target like the human dopamine receptor 2, with numerous pharmacology records, target similarity searches were not performed.

### Generating a merged list of compounds active against a target, ranked by bioactivity

**Figure 2:**
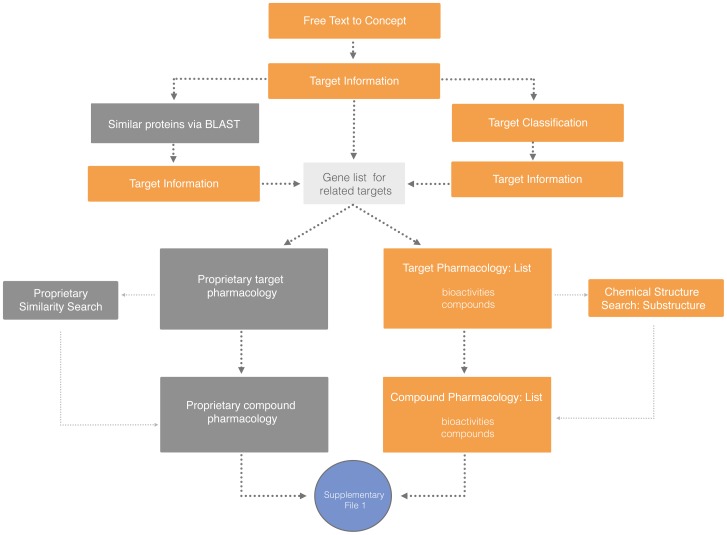
Use case A workflow. Schematic representation of the workflow for use case A. Starting with a free text search for the desired target(s), Uniprot AC identifiers, protein sequences and gene symbols are obtained using ‘Free Text to Concept’ and ‘Target Information’ API calls. A gene symbol list is obtained for targets from the same family (based on GO) using a ‘Target Classification’ API call. Alternatively, UniProt ACs obtained for related protein sequences via a BLAST search are used to get corresponding gene symbols using the ‘Target Information’ API call. Using this gene list, corresponding pharmacology records in the public domain are obtained via the ‘Pharmacology by Target’ API. In parallel, the gene symbol list is used to retrieve target pharmacology information in Thomson Reuters Integrity, World Drug Index, PharmaProjects, GVKBio GOSTAR, and Janssen pharmacology proprietary databases. Public pharmacology records (additional targets) for the retrieved compounds are then obtained using the ‘Pharmacology by compound’ API call with equivalent searches in Janssen pharmacology proprietary databases. If required, a structure similarity search is performed with the retrieved compounds to identify additional compounds, followed by another round of searches in Open PHACTS and proprietary databases as before. A Pipeline Pilot script was developed to run the above steps and produce an integrated list of compounds, activity data and target information from all databases. Proprietary components developed at Janssen were used to parse Janssen pharmacology data. All data processing was performed within the Pipeline Pilot framework.

A Pipeline Pilot workflow was created to provide a collection of targets, assay numbers, activity data, and chemical structure information from the databases mentioned above. The final steps of the workflow merge information per assay and data source, and sort the tabular results to present a ranked list of chemical compounds and their activities. In a facultative step, the workflow can also be programmed to search for similar chemical compounds and their pharmacological effects. This returns a complete activity profile for a comprehensive list of compounds of interest. A schematic representation of the workflow is shown in Fig. 2.

### Returning data for free text

Free text entered in the ‘Free Text to Concept’ API call can be used to find all corresponding concept URIs to enable usage of other API calls.

### Finding orthologues for a given target using free text

URIs for all orthologues of a given target were obtained using the ‘Free Text to Concept for Semantic Tag’ API call. The name of the target was used as free text input as above; the branch parameter was set to return concepts only from SwissProt data; and the tag concept parameter (i.e. the semantic type) was set to retrieve only those concepts tagged with ‘Amino Acid, Peptide, or Protein'.

### Returning data for a pathway

After choosing the pathway of interest on the WikiPathways website, the pathway can be used as input for queries with the Open PHACTS API in several different ways. Either the URI of the pathway is used directly (e.g. in the format of http://www.wikipathways.org/index.php/Pathway:WP1531) or the title or identifier of the pathway can be used in the ‘Free Text to Concept’ API call to retrieve a URI. Here, the branch parameter can be set to return concepts of WikiPathways only.

General information for the pathway such as the version of the data, the pathway title, and its description can be returned with the ‘Pathway Information’ API call.

A list of proteins and genes present in a pathway can be retrieved directly with ‘Pathway Information: Get Targets’. The API call results reflect the WikiPathways data, which can be either gene or protein URIs. The results can be used without further processing as input for target based API calls.

Pathways containing specific targets can be retrieved using ‘Pathways for Target: List’ API call. Either gene or protein URIs can be used as input.

### Creating heat-map and overlap representations of pharmacology data

To provide a better distribution for visualization, the activity values (for Potency, IC_50_, EC_50_, AC_50_, K_i_ and K_d_ endpoints) were transformed into their negative logarithmic Molar values (‘-logActivity values [molar]’). The same activity endpoints are available as ‘pCHEMBL values’ from the ChEMBL database, but in addition we also kept values with a relation different from ‘ = ’, but discarded the relation information for the following steps. For a binary representation (active: 1, inactive:0), a cutoff value of ‘-logActivity values [molar]’ of at least six was applied to determine active molecules.

A pivot table was generated to display bioactivities of compounds against multiple targets using the ‘Pivoting’ node in KNIME grouping rows by ‘Compound name’ and columns by ‘Target Name’. If several activity values are given for the same compound-target pair, only one value can be kept (e.g. a mean value or the most active value). In the case of the binary representation, ‘1’ (active) is chosen if an ambiguous classification is made. The resulting heat-maps were visualized with the HeatMap (JFreeChart) node in KNIME.

In order to detect compound specificity for single versus two or more targets within the pathway, an overlap table was generated. From the pivot table generated as above, the number of times a compound ‘hits’ a target was counted using the node ‘Column Aggregator’. The ‘Numeric row splitter’ node splits compounds hitting more than one target from those hitting just one. The former set was used to generate an overlap table.

### Retrieving pharmacology data for a target/compound and filtering options

The ‘Target Pharmacology: List’ API and ‘Compound Pharmacology: List’ API calls can be used to retrieve pharmacology data from ChEMBL for single protein targets and protein complexes containing the target. If only single protein targets are sought, the type is specified as target_type  =  single_protein in the API parameters. The pharmacology output is always filtered to exclude records where compound activity is unspecified. Values larger than 10^8^ are also removed to avoid potential data errors. The data can be filtered in many different ways, for example to return data for a specific activity (eg. IC_50_) or assay type (eg. binding or functional assays) or to only return agonists/activators or inhibitors/antagonists. Several different values can be requested in one call (e.g. IC_50_|EC_50_|AC_50_|K_i_|K_d_|Potency). Activity values can be limited by different cut-off parameters, for example by setting max-activity_value = 2000. The number of results for a given query can be retrieved with the ‘Target Pharmacology: Count’ or ‘Compound Pharmacology: Count’ API calls.

The data can be returned in one piece by using the parameter _pageSize = all. In cases which might return too many data points (e.g. several ten thousands), a smaller _pageSize parameter can be used, in combination with a loop overall result sets with the _page parameter.

### Finding Approved Drugs for an individual target or all targets in a pathway

The first approach uses the ‘Target Information’ API call where target URIs (gene or protein) are used as input. Compounds targeting this protein are derived from the DrugBank dataset where each molecule is labeled according to its type ('approved', 'biotech', 'experimental', 'illicit', 'investigational', 'nutraceutical', 'small Molecule', ‘withdrawn’). The resulting data are filtered for ‘Drug type = approved’. The second approach uses the ‘Target Pharmacology: List’ API call to find all compounds active against a given target based on ChEMBL records. These compound URIs are then used in the ‘Compound Information’ API call and results filtered for approved drugs as before. The search retrieves all approved drugs that have bioactivity against a given target, even if not approved for that target in DrugBank. The results from both approaches are merged.

### Retrieving Chemical Entities of Biological Interest (ChEBI) terms associated with a compound

ChEBI terms for a molecule are retrieved with the ‘Compound Classifications’ API call setting the tree parameter to ‘chebi’. The resulting data was restricted to classifications of the type “has role”, which includes the three sub-categories: ‘chemical role’, ‘biological role’, and ‘application’.

### Retrieving GO terms associated with a target

GO terms for a target can be retrieved using the ‘Target Classifications’ API call by setting the tree parameter to ‘go’. This returns classifications from the three branches of GO (cellular component, molecular function, and biological process). The resulting data was filtered for ‘biological process’.

### Retrieving positive and negative regulators of a pathway via GO terms

GO terms associated with the term ‘regulation of Vitamin D’ were obtained with the ‘Free text to Concept’ API call, the resulting data was restricted to ‘alternative’ exact match type, to include only GO terms. Children of these terms were retrieved using ‘Hierarchies: Child’ API call to enable separation of positive and negative regulators. Gene products associated with these GO terms were obtained using ‘Target Class Member: List’ API call

## Results

Three use case workflows were implemented to highlight different applications of the integrated Open PHACTS data. Use case A assembled a ranked list of compounds targeting the dopamine receptor D_2_ (DRD2) and then found related targets in both public and proprietary pharmacology databases to aid in the design of a new compound library for the dopamine receptor drug discovery program. Use case B identified compounds active against all targets in the Epidermal growth factor receptor (ErbB) signaling pathway that have a relevance to disease. Use case C evaluated established targets in the Vitamin D metabolism pathway and then expanded the scenario to view these targets in other contexts.

### Use case A: Comparison of existing public and proprietary pharmacology data for DRD2

The mesolimbic dopamine system is a central component of the brain reward circuit [28]. Pharmacological agents targeting dopaminergic neurotransmission have been clinically used in the management of several neurological and psychiatric disorders, including Parkinson's disease, schizophrenia, bipolar disorder, Huntington's disease, attention deficit hyperactivity disorder (ADHD), and Tourette's syndrome (reviewed by [29]). The physiological actions of dopamine are mediated by five distinct but closely related G protein-coupled receptors that are divided into two major groups: the D1-like (D_1_ and D_5_) and D2-like (D_2_, D_3_, D_4_) classes of dopamine receptors (DARs) on the basis of their structural, pharmacological, and biochemical properties [30], [31]. Of the five DARs and their variants, the DRD2 and its properties continue to be the most actively investigated because it is the main clinical target for antipsychotics and for the dopamine agonist treatment of Parkinson's disease [32]. Despite being one of the most validated targets for neuropsychiatric disorders, truly selective drugs for the DRD2 subtype have been hard to obtain due to high conservation of orthosteric binding sites among DARs and other GPCRs, leading to undesirable side-effects. As such, there has been tremendous effort to identify novel DRD2-selective ligands that will be useful not only as improved pharmacotherapeutic agents, but also to help define the function of D2-like receptor subtypes and as *in vitro* and *in vivo* imaging agents. We aimed to rank existing compounds known to target the DRD2 to aid in the design of a novel DRD2-targeted screening library.

#### Ranked list of public and proprietary compounds targeting DRD2

Our workflow (Fig. 2) for finding DRD2-targeted chemical matter (run in February 2014), identified 2278 ‘active’ organic compounds in Open PHACTS public repositories showing either % activity or IC_50_ values against the DRD2 (S1 File). Considering a cut-off of>50% for % activity values and -log(IC_50_) values>6, we identified 6194 bioactivity values; an additional 164 ‘inactive’ compounds are found with activity values below 50% or -log(IC_50_) values below 6 (Table 1). The same protocol identified 3148 organic compounds in patent reporting databases: Thomson Reuters Integrity monthly updates, World Drug Index quarterly reports, and PharmaProjects monthly updates were licensed from Thomson Reuters. 8959 additional compounds with over 50,000 activity and -log (IC_50_) data points are found in the in-house proprietary pharmacology screening database. The total number of compounds found is the sum of those found in the different sources as there is little overlap between them. This is because Open PHACTS/ChEMBL uses public information, Thomson Reuters uses patent information (often not published), and the in-house pharmacology databases use internal information (often not patented). Our workflow provides 2278 compounds that would have been missed altogether or difficult to find using approaches independent of Open PHACTS. In a facultative step, the workflow can also search for similar chemical compounds and their pharmacological effects, to present a complete activity profile for a comprehensive list of compounds of interest. Thus, using Open PHACTS we were able to produce a cohesive list of interesting DRD2-targeting compounds derived from heterogeneous data stored in multiple databases.

**Table 1:** Number of DRD2-targeted compounds found in different databases.

Activity Data Source	Number of Compounds found
Open PHACTS	2278 (active) +164 (inactive)
Patent Reporting Databases	3148
Janssen Compound Screening Databases	8959

Active compounds have % activity values>50% or -log(IC_50_) values>6.

The most interesting compounds have a high activity, or are reported in patent literature to act on the target of interest. They must also have little reported activity on other targets. Conversely, the least interesting compounds have low or no reported activity on targets of interest and have higher reported activity on other targets. This sorting allows a more efficient processing of tables that sometimes contain data on several hundreds of compounds. A Pipeline Pilot script running all the steps described above automatically produces a relevant listing of compounds, activity data, and target information in under an hour, making the process of looking for compounds for new targets and target families a simple and reproducible task. The above script allows control of the different process steps, and has been successfully used at Janssen to support various drug discovery projects.

Finally, programmatic access to the individual data sources previously required a specific case by case approach: for example, access to biological activity data from ChEMBL was via a locally installed MySQL database, from DrugBank from a copy of the XML, from GVKBio GOSTAR from a remotely installed Oracle database, from Thomson Reuters from a tab-delimited text file, and from the in-house pharmacology database from a local server-based Oracle database. Searching the different databases for target information was done mostly manually, where information had to be carefully assembled for each target in each database and the process repeated for each request for new target information. By using Open PHACTS, data from ChEMBL and DrugBank could be retrieved from a single source, reducing the effort needed for data integration. The custom Pipeline Pilot Open PHACTS component library enabled access to the databases in Open PHACTS, on par with components already in use for proprietary databases, thereby allowing a true integration of all available pharmacology data in one protocol. The workflows for retrieving the data from the different data sources are depicted in a Pipeline Pilot screenshot S1 Fig.

This example illustrates the benefit of accessing the Open PHACTS data in the competitive Pharmaceutical research environment, even for well-known targets that have already been extensively studied.

### Use case B: Compounds active against targets in the ErbB signaling pathway and their disease relevance

Epidermal growth factor receptors (known as ErbB) are receptor tyrosine kinases consisting of four members: ErbB1/EGFR, ErbB2/HER2, ErbB3 (HER3), and ErbB4 (HER4). Members of the EGF family of growth factors (e.g. EGF, neuregulins), are natural ErbB receptor ligands which upon binding induce homo- or heterodimerization of the receptor and subsequent activation of intrinsic kinase activity [33]. Different ErbB heteromers activate different downstream signaling pathways (http://www.wikipathways.org/index.php/Pathway:WP673): mitogen-activated protein kinase (MAPK) signaling and phosphatidylinositol 3-kinase (PI3K)-AKT pathway, SRC tyrosine kinase pathway, signal transducer and activator of transcription proteins (STATs), and mammalian target of rapamycin(mTor) pathway [33]. Upon activation of different branches of the ErbB signaling network, different responses are triggered ranging from cell division to death, motility to adhesion. Insufficient ErbB signaling in humans is associated with the development of neurodegenerative diseases, such as multiple sclerosis and Alzheimer's disease [34]. ErbB-1 and ErbB-2 are found in many human cancers and [35], [36] their excessive signaling is associated with the development and malignancy of these tumors. Accordingly, the ErbB receptor family with their most prominent members EGFR and HER-2 represent validated targets for anti-cancer therapy, and anti-ErbB monoclonal antibodies (e.g. cetuximab, panitumumab, and trastuzumab) and tyrosine kinase inhibitors (gefitinib, erlotinib, and lapatinib) have now been approved for the treatment of advanced colorectal cancer, squamous cell carcinoma of the head and neck, advanced non-small-cell lung cancer, as well as pancreatic and breast cancer [33].

However, current therapy treats only a subset of patients carrying specific mutations and even within this population, tumor resistance is common. Identification of specific protein targets involved in ErbB-mediated cancer development is confounded by the multiplicity of pathways activated by ErbB receptors and the existence of more than 100 potential protein binding partners identified by large-scale phosphoproteomic screening [37]. As members of the ErbB receptor family cooperate in signal transduction and malignant transformation, the concurrent inhibition of two or more receptors or specific heteromeric ErbB family receptor complexes may yield the next generation targeted therapies. However, only a small proportion of publicly available bioactivity data reports on the activation of ErbB oligomers. In many cases, the exact mechanism of ligand-protein binding and protein activation is simply not known and bioactivity of small molecules is tested on single proteins only. This leads to challenges for structure-based drug design and interpretation of pharmacological data. As such, understanding the role of receptor oligomers in the ErbB signaling pathway is invaluable for the purpose of drug discovery.

#### Pathway targets and pharmacology

**Figure 3:**
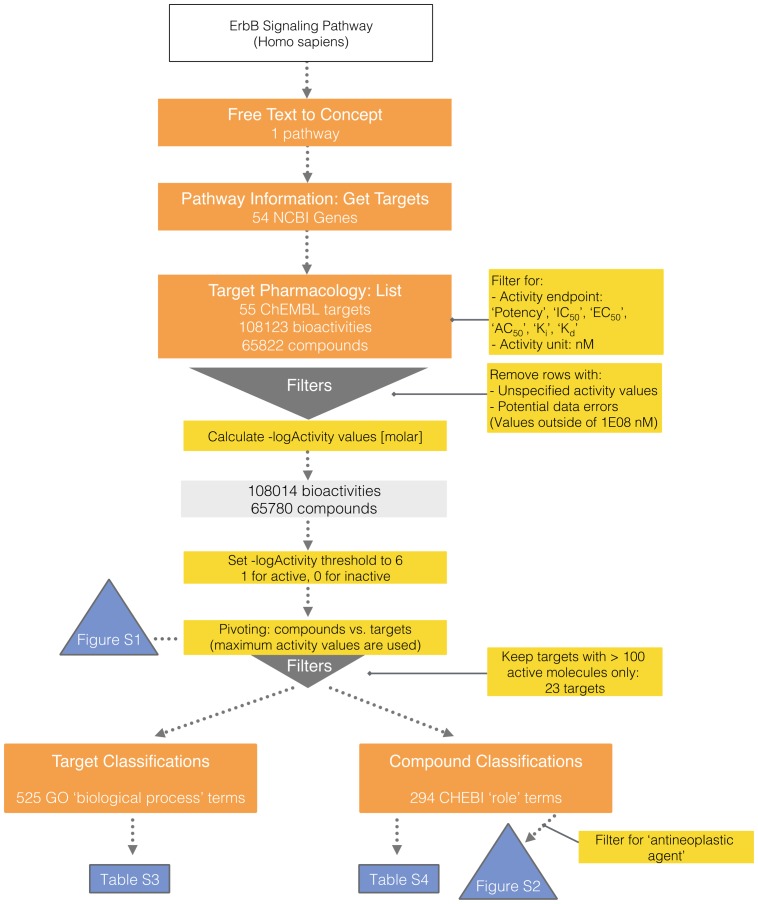
case B workflow. Open PHACTS v 1.3 API calls are shown in orange boxes along with the results obtained. Bioactivity filters and other data processing operations are shown in yellow boxes with results obtained in light grey boxes. Blue colored boxes show results included in the manuscript. Compound pharmacology at the pathway level was retrieved by consecutive execution of the API calls ‘Pathway Information: Get targets’ and ‘Target Pharmacology: List’ - the latter includes a filtering for desired activity endpoints and units - and other filtering, transformation, and normalization steps: transformation into ‘- logActivity values [molar]’, setting a threshold for binary representation, and subsequent filtering by keeping only the max. activity value for each compound/target pair. Retrieving GO annotations for a list of targets, and ChEBI annotations for compounds that have been tested against those targets was achieved by using the API calls ‘Target Classifications’ and ‘Compound Classifications’ and subsequent restriction to terms of the type ‘biological process’ and ‘has role’, respectively.

In total, 54 NCBI Gene IDs were retrieved as targets from the ErbB signaling pathway. Of those, only 35 single proteins returned pharmacological data with the applied bioactivity filters. Additionally, data for 12 protein families, 5 protein complexes, 2 protein-protein interactions and one chimeric protein containing a target from the pathway were retrieved, increasing the total number of targets to 55. While a pharmacology query without any filters would retrieve nearly 150,000 data points, filtering reduced the data to 108,014 bioactivities and 65,780 unique compounds (see Fig. 3). Using the pChEMBL values to filter bioactivities led to a significantly lower number of records as compared to -logActivity values: 53 targets, 65,817 bioactivity endpoints and 43,255 unique compounds. The pChEMBL filter restricts data to those that are equal to a specific value. Values that are reported to be ‘greater than’ or ‘less than’ will therefore be missing in the final data set. Consequently, -logActivity values appear to be a valid approach to generate data sets of bioactivity measures that span a larger range of values.

To compare the pharmacological data across different targets, each compound/target pair was represented by only one activity point, keeping the most active value in cases where several measurements were reported, and a cutoff was set for separating active from inactive compounds. A heat map representation of the compound/target space was retrieved for these binary representations (S2 Fig.). Protein targets with a greater number of measurements (having a larger portion of red/blue bars) can be distinguished from those with a lower number of activity data points (having a large portion of grey bars). For instance, targets: Cellular tumor antigen p53 (CHEMBL4096, P04637), MAP kinase ERK2 (CHEMBL4040, P28482), Epidermal growth factor receptor ErbB1 (CHEMBL203, P00533), and FK506 binding protein 12 (CHEMBL2842, P42345), have the highest numbers of unique measurements (sum of unique active and inactive compounds), 36,075, 14,572, 5,028, and 4,572, respectively. In addition, one can identify targets with a higher number of unique active compounds (setting the cutoff at 6), i.e. 3,670 for p53, and 2,268 for ErbB1 (see Table 2). By reducing the target/compound space to representative activity points and choosing a binary representation, easier visualization of large data collections is enabled. However, additional information on the concrete bioactivity might be desirable in cases where compounds possess activity values close to the chosen cutoff.

**Table 2:** List of 23 targets (possessing more than 100 active compounds) with their ChEMBL Target IDs, target names, target types, and the number of active and inactive compounds that have been tested on those targets (considering a threshold of 6).

ChEMBL target ID	Target Name	Target Type	number of actives	number of inactives
CHEMBL4096	Cellular tumor antigen p53	Single Protein	3670	32405
CHEMBL203	Epidermal growth factor receptor erbB1	Single Protein	2268	2760
CHEMBL267	Tyrosine-protein kinase SRC	Single Protein	1567	2243
CHEMBL262	Glycogen synthase kinase-3 beta	Single Protein	1536	1547
CHEMBL2842	FK506 binding protein 12	Single Protein	1328	3244
CHEMBL4040	MAP kinase ERK2	Single Protein	1230	13342
CHEMBL1862	Tyrosine-protein kinase ABL	Single Protein	1077	614
CHEMBL1824	Receptor protein-tyrosine kinase erbB-2	Single Protein	964	1214
CHEMBL2276	c-Jun N-terminal kinase 1	Single Protein	607	1215
CHEMBL299	Protein kinase C alpha	Single Protein	528	483
CHEMBL3587	Dual specificity mitogen-activated protein kinase kinase 1	Single Protein	444	475
CHEMBL1907601	Cyclin-dependent kinase 4/cyclin D1	Protein Complex	381	379
CHEMBL4501	Ribosomal protein S6 kinase 1	Single Protein	299	582
CHEMBL2695	Focal adhesion kinase 1	Single Protein	230	825
CHEMBL4816	Serine/threonine-protein kinase AKT3	Single Protein	163	876
CHEMBL2095188	Glycogen synthase kinase-3	Protein Family	158	201
CHEMBL3663	Growth factor receptor-bound protein 2	Single Protein	145	168
CHEMBL4482	Serine/threonine-protein kinase PAK 4	Single Protein	129	1016
CHEMBL5023	p53-binding protein Mdm-2	Single Protein	127	307
CHEMBL2095942	Cyclin-dependent kinase 4/cyclin D	Protein Complex	113	42
CHEMBL1907611	Tumour suppressor p53/oncoprotein Mdm2	Protein Protein Interaction	107	252
CHEMBL2096618	Bcr/Abl fusion protein	Chimeric Protein	107	106
CHEMBL3009	Receptor protein-tyrosine kinase erbB-4	Single Protein	102	683

Apart from necessary filtering and normalization steps that limit the full illustration of the target space, we also recognized a lack of reliable compound bioactivity data specifically targeting oligomeric proteins in the pathway. For example, in ChEMBL_v17, the target ‘Epidermal growth factor receptor and ErbB2 (HER1 and HER2)’ is classified as being a ‘protein family’ (CHEMBL2111431, P00533 and P04626) with 115 IC_50_ bioactivity endpoints. Inspecting the underlying assay descriptions however reveals the inclusion of compounds targeting either ErbB1, ErbB2, both proteins, or in some cases even upstream targets. For the sake of data completeness, we retained all target types in the query, but we advise to always go back to the original primary literature source and study the bioassay setup in order to make sure which effect was actually measured and if the data is reliable in cases where data is assigned to other target types than ‘single protein’.

#### Studying targets related to certain diseases

Determining the targets related to cancer or neurodegenerative diseases was accomplished by evaluating the GO [22], [23] annotations. The ‘biological process’ terms were extracted for the 23 protein targets (possessing at least 100 active compounds): 525 different (unique) annotations, with Glycogen synthase kinase-3 (CHEMBL2095188, P49840 and P49841; 93 annotations), and p53 (CHEMBL4096, P04637; 86 annotations) having the highest number of different annotation terms. The GO term most frequently associated with the 23 targets was ‘innate immune response’ (GO_0045087; annotated to 16 targets). Interestingly, brain immune cells (microglia) seem to play a major role in the development and progress of neurodegenerative diseases such as Alzheimer's disease [38], [39]. Other frequent terms, which appear interesting in the context of cancer include: ‘negative regulation of apoptotic process’ (GO_0043066; annotated to 9 targets), ‘positive regulation of cell proliferation’ (GO_0008284; 7 targets), ‘cell division’ (GO_0051301; 6 targets), ‘apoptotic process’ (GO_0006915; 5 targets), and ‘positive regulation of apoptotic process’ (GO_0043065; 5 targets). The information gained by such analyses can guide the selection of targets to be studied more thoroughly, in the search for novel therapeutic treatment opportunities, especially if multi-targeted therapies are in the focus of research. (A list of all GO ‘biological process’ terms that have been annotated to at least 5 of the 23 prioritized targets and ChEMBL target IDs of those targets can be found in the S3 Table.)

#### Studying compounds related to certain diseases

In parallel to the identification of GO terms for the targets, we enriched the compounds with the addition of ChEBI terms [17], [18], [19]. In total, 294 different ChEBI ‘roles’ (including the three sub-categories: ‘chemical role’, ‘biological role’, and ‘application’) have been annotated to 1036 different compounds targeting the 23 prioritized targets. Unfortunately, only a minor proportion of compounds (approximately 1,6% in this use case) possess ChEBI annotations although they are of very high quality as each entry in the database is manually annotated by experts [17]. 49 of the 294 different (unique) ChEBI terms have been annotated to at least 6 different compounds (see Suppl. Section, S4 Table). The ChEBI term ‘antineoplastic agent (ChEBI_35610)’ appears the most frequently, with annotations to 79 different compounds. We assessed these active compounds using a binary heatmap representation (see S3 Fig.) and found the targets: Tyrosine-protein kinase ABL (CHEMBL1862, P00519; 18 active compounds), Epidermal growth factor receptor ErbB1 (CHEMBL203, P00533; 15 active compounds), and Tyrosine-protein kinase SRC (CHEMBL267, P12931; 10 active compounds) with the highest numbers of active measurements. Compounds comprising a pharmacological pattern corresponding to that (activity on CHEMBL1862, CHEMBL203, and CHEMBL267) and possessing the ChEBI annotation term ‘antineoplastic agent’ include: Erlotinib, Lapatinib, Bosutinib, Vandetanib, Sunitinib, Masitinib, Canertinib, and Sprycel. It appears interesting to experimentally test other compounds with the same ChEBI term against those three targets, especially if they possess a similar chemical structure like the compounds/drugs mentioned before. S2 File gives the names of the 79 compounds, their CHEMBL compound IDs, and the previously determined active/inactive result according to our cut-off for active molecules.

However - like all hand-curated resources - ChEBI is biased towards its annotation criteria, which in that case are already approved drugs. Thus, to date it serves best for filtering out drugs related to a certain disease. As the ChEBI database and ontology is instantly growing, it will become a more comprehensive and increasingly reliable and useful resource.

Using our Open PHACTS workflow, we could answer research questions related to complex regulatory pathways with a large number of druggable targets and requiring data from multiple sources. With an expansion of the data sources available in the next release of the Open PHACTS API (version 1.4), which will include more information on the distribution of targets in tissues and changes in relation to disease, more refinement of the antineoplastic agents found in our analyses will be possible.

### Use case C: Broadening the therapeutic opportunities from the Vitamin D pathway

1,25(OH)_2_D_3_ or calcitriol, the biologically active form of vitamin D [40], is an important hormone that is critically required for the maintenance of mineral homeostasis and structural integrity of bones by facilitating calcium absorption from the gut and by direct action on osteoblasts, the bone forming cells [41]. Apart from its classical actions on the gut and bone, calcitriol and its synthetic analogues also possess potent anti-proliferative, differentiative and immunomodulatory activities (reviewed by [46]). These pleiotropic effects are mediated through vitamin D receptor (VDR), a ligand-dependent transcription factor that belongs to the superfamily of steroid/thyroid hormone/retinoid nuclear receptors [42]. This has set the stage for therapeutic exploitation of synthetic VDR ligands for the treatment of various inflammatory indications and cancer [43], [44], [45], [46], [47], [48]. However, the use of VDR ligands for these indications in the clinic is limited by their major dose-related side effect, viz., hypercalcemia/hypercalciuria. Therefore there has been tremendous interest in generating newer vitamin D analogues that retain the desired therapeutic activity but with less toxic (calcemic) side effects.

Prior to reaching the nuclear VDR, calcitriol interacts with several key proteins, the serum vitamin D binding protein (DBP), the vitamin D-activating enzyme (CYP27B1), and the catabolic enzyme 24-hydroxylase (CYP24A1). The latter two enzymes are expressed and differentially regulated in VDR-expressing target tissues, providing a means for tissue-specific actions of VDR ligands. Affinity for the DBP is another means to control circulating calcitriol levels. The unique actions of calcitriol and its analogues thus result from their combined interactions with several key proteins in the Vitamin D pathway (http://www.wikipathways.org/index.php/Pathway:WP1531). Better understanding of these interactions and a pathway-focused approach will facilitate the design of a new generation of vitamin D analogues with a desired interaction profile against pathway components, resulting in improved therapeutic indices. Knowledge of the appropriate compound evaluation methodologies is also important to ensure that the desired bioactivity profile is being retained during chemical optimization stages. Finally, information about how the pathway is regulated, identifying novel points for therapeutic intervention, and estimating the impact of modulating these targets could allow alternative therapeutic strategies. Accordingly, our Open PHACTS workflows were designed to collect the above information and identify drug discovery opportunities in the Vitamin D metabolism pathway.

#### Pathway targets and pharmacology

**Figure 4:**
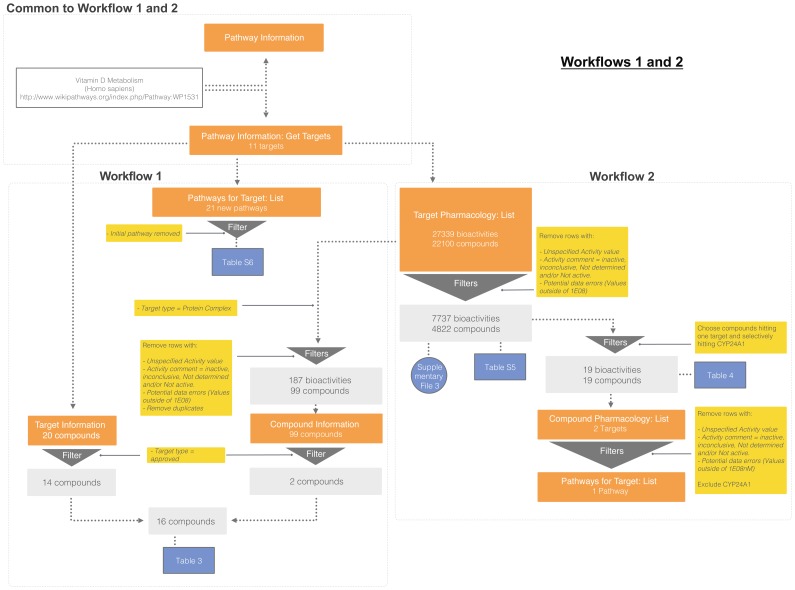
Use case C workflows 1 and 2. Open PHACTS v 1.3 API calls are shown in orange boxes along with the results obtained. Bioactivity filters and other data processing operations are shown in yellow boxes with results obtained in light grey boxes. Blue colored boxes show results included in the manuscript. Sample input URLs are shown in S2 Table. For workflow 1, a description of the pathway and targets contained were obtained using the ‘Pathway information’ and ‘Pathway Information: Get targets’ API calls. Other pathways where these targets are present were obtained using ‘Pathways for Target: List’ API call. Approved drugs against single protein targets were obtained using ‘Target Information’ API call by specifying target type - approved. Compounds tested against all targets in the pathway were retrieved using ‘Target Pharmacology: List’ API call. Approved drugs targeting protein complexes (containing any member of the pathway) were identified by filtering for protein complexes and ‘approved’ target type via the ‘Compound Information’ API call. For workflow 2, compounds hitting CYP24A1 from the previous results were used as input to find additional targets using the ‘Compound Pharmacology: List’ API. Additional pathways containing these new targets were obtained using ‘Pathways for Target: List’ API.

The pathway data obtained (from workflows 1 and 2, represented in Fig. 4) afforded several insights into the Vitamin D metabolism pathway; names of targets, number of compounds tested, their specificity for these targets and approved drugs in the pathway are shown in Table 3 and S5 Table. Other pathways where these targets are present are shown in S6 Table. From these data we see that out of the 10 targets in the pathway, 4139 unique compounds are reported to have activity against the target VDR and 545 for RXR-alpha, compared to 323 compounds for all the remaining targets combined (S3 File). This provides a quick overview on which targets in the pathway have been the focus of small molecule modulatory approaches and the ‘undruggable’ targets are identified - parathyroid hormone and CYP2R1/Vit D- 25 hydroxylase. Existing approved drugs in DrugBank for single protein targets are obtained via the ‘Target Information’ API. To complement this information, we obtained pharmacology data from ChEMBL for protein complexes consisting of pathway components using the ‘Target Pharmacology’ API. Indeed, no approved drugs are listed in DrugBank 3.0 for DHCR7; however our workflow retrieves Tamoxifen and Doxorubicin as they target the anti-estrogen binding site (AEBS), a protein complex comprising DHCR7 and D8-D7 sterol isomerase [49]. The integration of two disparate pharmacology databases (DrugBank and ChEMBL) provides a more complete listing of all approved drugs that have potent activity against any target in the pathway, whether it is a single protein or part of a complex. Thus, in one workflow, we could quickly assess the previously published chemical space of a pathway of interest.

**Table 3:** List of targets, compounds and approved drugs in Vitamin D metabolism pathway obtained from Workflow 1.

No.	UniProt Accession	Name	Active compounds (bioactivities)	Approved drugs obtained via 2 methods		Comment
				Target information API (DrugBank 3.0)	Target Pharmacology API (ChEMBL_16)	
**1**	Q07973	CYP24A1 cytochrome P450	26 (30)	0		
**2**	Q548T3	CYP27B1 cytochrome P450	1 (1)	Ergocalciferol		
				Calcidiol		
				Calcitriol		
**3**	Q02318	Sterol 26-hydroxylase, mitochondrial	5 (5)	Cholecalciferol		
						
**4**	P02774	Vitamin D-binding protein	39 (112)	0		
**5**	Q9UBM7	DHCR7 7-dehydrocholesterol reductase	4 (5)	NADH		
	Q15125, Q9UBM7	AEBS	66 (122)	0	Tamoxifen, Doxorubicin	Target is protein complex AEBS (made of DHCR7 and D8-D7 sterol isomerase). Not listed in DrugBank 3.0
**7**	Q6VVX0	CYP2R1 cytochrome P450	0 (0)	Cholecalciferol, Ergocalciferol		
**8**	P01270	PTH parathyroid hormone	0 (0)	0		
**9**	P19793	RXRA retinoid X receptor, alpha	545 (1274)	Alitretinoin		
				Acitretin		
				Adapalene		
	P37231, P19793	RXR alpha/PPAR gamma	33 (65)	0	0	
**10**	P28702	RXRB retinoid X receptor, beta	149 (261)	Alitretinoin		
				Bexarotene		
				Acitretin		
				Adapalene		
				Tazarotene		
				Tretinoin		
**11**	P11473	VDR vitamin D (1,25- dihydroxyvitamin D3) receptor	4139 (5918)	Calcipotriol		
				Calcitriol		
				Ergocalciferol		
				Paricalcitol		
				Dihydrotachysterol		
				Calcidiol		

#### CYP24A1 as a therapeutic target

The pathway pharmacology data clearly show that the majority of efforts have been focused on targeting the VDR directly (Table 3). Targets for novel therapeutic strategies to enhance VDR activation could lie upstream of ligand-receptor binding, at the level of calcitriol catabolism by CYP24A1 [50] or transport by Vitamin D- binding protein or DBP [51]. CYP24A1 is the major catabolic enzyme of calcitriol converting it to less active calcitroic acid [52], so selectively inhibiting this enzyme can be expected to raise the circulating levels of the hormone or its analogues. Therefore, using Workflow 2 (represented in Fig. 4) we looked for compounds with inhibitory activity against CYP24A1 and found 25 unique compounds, of which 12 have IC_50_ <10 uM (Table 4).

**Table 4:** Compounds active against CYP24A1 obtained from Workflow 2.

No.	Compound name	Assay Description	Activity Type	Value	Unit	pChembl	Active against other targets in pathway?	Other targets in general?
**1**	**4'-Chloro-N-[(2R)-2-(1H-imidazol-1-yl)-2-phenylethyl]-4-biphenylcarboxamide**	**Inhibition of CYP24A1**	**IC50**	**15**	**nM**	**7.82**	**No**	**No**
**2**	**1,3-cyclohexanediol, 4-methylene-5-[(2E)-2-[(1R,3aS,7aR)-octahydro-7a-methyl-1-[(1R)-1-methyl-3-(phenylsulfonyl)propyl]-4H-inden-4-ylidene]ethylidene]-, (1R,3S,5Z)-**	**Inhibition of human CYP24 hydroxylase expressed in V79 cells**	**IC50**	**28**	**nM**	**7.55**	**No**	**No**
**3**	**N-(2-imidazol-1-yl-2-phenyl-ethyl)-4-[(E)-styryl]benzamide**	**Inhibition of human recombinant CYP24A1 expressed in chinese hamster V79 cells using [3H-1-beta]calcitriol after 60 mins by scintillation counting**	**IC50**	**300**	**nM**	**6.52**	**No**	**No**
**4**	**6-methoxy-2-(2-methylbenzyl)-3,4-dihydronaphthalen-1(2H)-one**	**Inhibition of CYP24A1 expressed in CHO cells**	**IC50**	**900**	**nM**	**6.05**	**No**	**Liver microsomes, ADMET (CYP26A1)**
**5**	**N-[2-(4-fluorophenyl)-2-imidazol-1-yl-ethyl]benzofuran-2-carboxamide**	**Inhibition of human recombinant CYP24A1 expressed in chinese hamster V79 cells using [3H-1-beta]calcitriol after 60 mins by scintillation counting**	**IC50**	**2800**	**nM**	**5.55**	**No**	**No**
**6**	**N-(2-imidazol-1-yl-2-phenyl-ethyl)benzofuran-2-carboxamide**	**Inhibition of human recombinant CYP24A1 expressed in chinese hamster V79 cells using [3H-1-beta]calcitriol after 60 mins by scintillation counting**	**IC50**	**4000**	**nM**	**5.40**	**No**	**No**
**7**	**N-(2-imidazol-1-yl-2-phenyl-ethyl)-5-nitro-benzofuran-2-carboxamide**	**Inhibition of human recombinant CYP24A1 expressed in chinese hamster V79 cells using [3H-1-beta]calcitriol after 60 mins by scintillation counting**	**IC50**	**6400**	**nM**	**5.19**	**No**	**No**
**8**	4'-chloro-N-[2-(1H-imidazol-1-yl)-2-phenylethyl]biphenyl-4-carboxamide	Inhibition of CYP24 in human keratinocytes	IC50	15	nM	7.82	25-hydroxyvitamin D-1 alpha hydroxylase, mitochondrial	No
**9**	1-[4-(4-{[(2S,4R)-2-(2,4-Dichlorophenyl)-2-(1H-imidazol-1-ylmethyl)-1,3-dioxolan-4-yl]methoxy}phenyl)-1-piperazinyl]ethanone	Inhibition of CYP24A1 in human epidermal keratinocytes	IC50	126	nM	6.90	RXRA, VDR	340 different targets (ketoconazole)
**10**	2-(2-Ethylbenzyl)-6-methoxy-3,4-dihydro-1(2H)-naphthalenone	Inhibition of CYP24A1 expressed in CHO cells	IC50	1920	nM	5.72	Sterol 26-hydroxylase, mitochondrial	No
**11**	6-Methoxy-2-[2-(trifluoromethyl)benzyl]-3,4-dihydro-1(2H)-naphthalenone	Inhibition of CYP24A1 expressed in CHO cells	IC50	2080	nM	5.68	Sterol 26-hydroxylase, mitochondrial	No
**12**	(2E)-6-Methoxy-2-{2-[(E)-2-phenylvinyl]benzylidene}-3,4-dihydro-1(2H)-naphthalenone	Inhibition of CYP24A1 expressed in CHO cells	IC50	5080	nM	5.29	Sterol 26-hydroxylase, mitochondrial	No

Compounds 1–7 ranked according to potency (in bold) have no activity against additional targets based on polypharmacology records, whereas compounds 8–12 inhibit calcitriol activating enzymes, VDR and RXRA.

Five of these compounds have potent activity against two other critical targets in the pathway, CYP27A1 and CYP27B1, the key activating enzymes producing calcitriol. One of these is ketoconazole, an approved drug for fungal infections that has been extensively tested against a variety of other targets in primary HTS and ADMET assays. The remaining seven compounds (five azoles and two non-azoles) could serve as starting points for selective CYP24A1 inhibition strategies given the lack of polypharmacology data and potential for off-target effects (Table 4). In addition, our data show that CYP24A1 does not have a known role in pathways other than Vitamin D metabolism (S6 Table), so inhibiting this enzyme should not affect substrates other than calcitriol (or its analogues), resulting in the desired prolongation of VDR activation. Therefore, a drug combination strategy of inhibiting CYP24A1 with one of the above compounds, while activating VDR with the natural ligand or an analogue may be considered as a valid approach to enhance VDR signaling [53]. Alternatively, evaluating a compound's sensitivity to CYP24A1, in parallel to VDR activation would optimize medicinal chemistry efforts to synthesize improved VDR ligands with better metabolic stability. Our polypharmacology (S3 File) data retrieved a vitamin D analogue (CHEMBL564855) with considerably less sensitivity to CYP24A1 catabolism (binding affinity to human CYP24A1 relative to calcitriol  = 2%) compared to the natural hormone while having high binding affinity to VDR (binding affinity to bovine thymus VDR relative to calcitriol  = 180%), that could serve as a starting point for this approach [54].

#### Evaluating compound affinity for VDR and DBP orthologues

**Figure 5:**
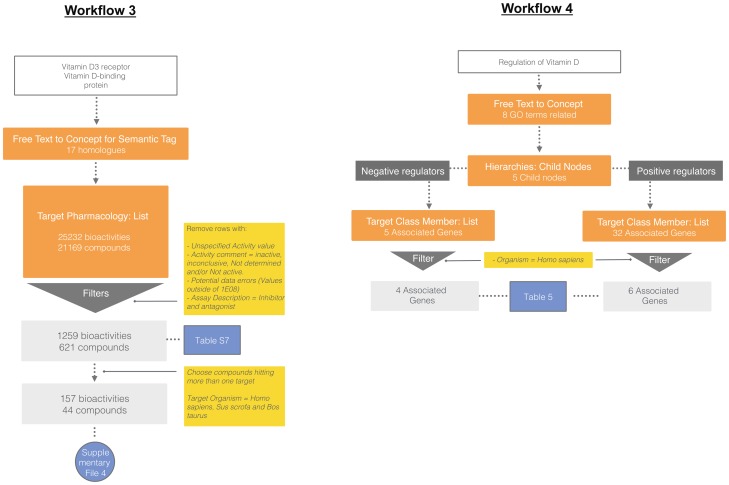
Use case C workflows 3 and 4. Open PHACTS v 1.3 API calls are shown in orange boxes along with the results obtained. Bioactivity filters and other operations are shown in yellow boxes. Results obtained after these operations are shown in light grey boxes. Blue colored boxes show results included in the manuscript. Sample input URLs are shown in S2 Table. For workflow 3, Urls for all species orthologues of a given target were obtained using ‘Free Text to Concept for Semantic Tag’ API. Pharmacology data for these orthologues was obtained using ‘Target Pharmacology: List’ API. Data was limited to compounds tested in binding affinity assays from bovine, porcine and human in both VDR and DBP by applying appropriate filters in KNIME. For workflow 4, GO terms related to ‘Regulation of Vitamin D’ were obtained using the ‘Free Text to Concept’ API. Children of these GO terms were obtained using ‘Hierarchies: Child Nodes’ API. The data were sorted by positive/negative regulation. Gene products associated with these GO terms were obtained using ‘Target Class Member: List’ API.

There is considerable Structure Activity Relationship (SAR) data on the VDR as compared to the DBP, although the latter is a critical determinant of Vitamin D analogue availability *in vivo*. However, of the 669 human VDR-activating compounds retrieved, only two have been tested for human DBP binding (S3 File). The amino acid sequence of the VDR ligand-binding domain (residues 192–427) is highly conserved, with the bovine and porcine orthologues sharing 96% and 97% similarity, respectively, with that of the human VDR, allowing comparisons to be made for binding assays. We therefore expanded our search to orthologues of these two targets (S7 Table) to retrieve compounds with binding affinity data for VDR from three species (workflow 3 represented in Fig. 5). We identified 35 such compounds that also had binding affinity data for human DBP; a more reasonable number for SAR analysis (S4 File). Preliminary observations show that most compounds involve modifications of side chain or A-ring structures but a more limited set of four compounds are non-steroidal structures. Interestingly, these newer analogues have no affinity for DBP compared to the classical steroidal analogues but are capable of binding VDR with moderate affinity and moreover show lower calcemic activity [55]. It is reasonable to speculate that designing analogues with lower DBP binding will enable higher target tissue concentration and lower their lower calcemic effects *in vivo*. Indeed several reports describing other non-steroidal Vitamin D analogues can be found in the literature [56], [57], [58], [59], [60]. However, as they have been explicitly tested for DBP binding, they could not be included in the SAR analysis set for non-secosteroidal analogues.

#### Regulation of the pathway

We used Gene Ontology (GO) annotations [22] for a preliminary assessment of factors that regulate Vitamin D signaling in general, and those that specifically regulate key enzymes in the pathway (Workflow 4 represented in Fig. 5). In addition to external factors, we identified pathway components that regulate Vitamin D signaling via inherent feedback loops. For example, CYP24A1, the main catabolic enzyme of 1,25(OH)_2_D_3_ is upregulated by the VDR, providing an efficient negative feedback loop to terminate calcitriol actions in normal conditions (Table 5). Conversely, abnormally elevated CYP24A1 in certain disease states, such as hypophosphatemia [61], [62] and certain types of cancer [63] associates with decreased vitamin D status and with vitamin D resistance. CYP24A1 may thus be a predictive marker of 1,25(OH)_2_D_3_ efficacy as an adjunctive therapy in patients with cancer. Next, we see that the transcription factors SNAIL1 and SNAIL2 repress Vitamin D signaling by inhibiting VDR expression (Table 5). Interestingly, these factors have been shown to be elevated in several types of cancers and thought to be the mechanism by which these cancers are resistant to tumor suppressor action by endogenous 1,25(OH)_2_D_3_ [64], [65], [66]. Patients with high levels of SNAIL1 and SNAIL2 can be expected to have lower VDR expression and, therefore, will be poor responders to anti-cancer therapy with 1,25(OH)_2_D_3_ or its analogs. Thus, tumor expression of SNAIL1 and SNAIL2 could also be used as biomarkers of adequacy for this type of therapy [67]. The GO annotations extended our knowledge of the interactions between pathway components to gain valuable insights into the mechanisms for feedback regulation, as well as identify potential biomarkers for selecting tumors most likely to respond to Vitamin D analogue therapy.

**Table 5:** Regulators of Vitamin D signaling obtained from Workflow 3.

GO terms associated with Vitamin D regulation		Associated Genes (Human)		In pathway?
Parents	Children	UniProt Accession	TargetName/Gen Name	
GO:0010979 regulation of vitamin D 24-hydroxylase activity	**GO:0010980 positive regulation of vitamin D 24-hydroxylase activity**	O15528	25-hydroxyvitamin D-1 alpha hydroxylase, mitochondrial	YES
		**P11473**	**Vitamin D3 receptor**	**YES**
		Q9GZV9	Fibroblast growth factor 23	NO
GO:0060556 regulation of vitamin D biosynthetic process	GO:0060557 **positive** regulation of vitamin D biosynthetic process	P01579	Interferon gamma	NO
		P01375	Tumor necrosis factor	NO
GO:0070562 regulation of vitamin D receptor signaling pathway	GO:0070564 **positive** regulation of vitamin D receptor signaling pathway	O15528	25-hydroxyvitamin D-1 alpha hydroxylase, mitochondrial	YES
		Q13573	SNW domain-containing protein 1	NO
GO:0060556 regulation of vitamin D biosynthetic process	GO:0010957 **negative** regulation of vitamin D biosynthetic process	O43623	**Zinc finger protein SNAI2**	NO
		O95863	**Zinc finger protein SNAI1**	NO
		P19838	Nuclear factor NF-kappa-B p105 subunit	NO
		Q99684	Zinc finger protein Gfi-1	NO
GO:0070562 regulation of vitamin D receptor signaling pathway	GO:0070563 **negative** regulation of vitamin D receptor signaling pathway	O43623	**Zinc finger protein SNAI2**	NO

Terms in bold are discussed in the text.

In conclusion, knowledge of the Vitamin D metabolism pathway obtained through these workflows supports and informs on a multi-pronged drug discovery approach, wherein properties like DBP binding and sensitivity to CYP24A1 catabolism are evaluated in parallel using the appropriate bioassays, rather than focusing on VDR activation alone. An effective analogue should potently activate VDR, be resistant to catabolism by CYP24A1 and have low affinity for DBP. Alternatively, co-administration with a selective CYP24A1 inhibitor could also extend analogue lifetime. Most tissues express VDR, so tissue-specific actions of VDR ligands are instead governed by differential expression and regulation of CYP27B1, which permits localized synthesis of additional calcitriol, and CYP24A1, which inactivates the hormone. Tissue expression profiles as well as interacting proteins for a given target can be obtained in future versions of the Open PHACTS Discovery Platform with the incorporation of neXtProt data and tissue ontologies, thereby enabling a better prediction of 1,25(OH)_2_D_3_ analogue efficiency in different cellular contexts.

## Conclusions and Future Directions

The Open PHACTS Discovery Platform makes available the data needed to answer a wide range of questions applicable to pharmaceutical research by broadly covering critical aspects of chemistry and biology. A multitude of potential use cases of the Open PHACTS Discovery Platform can be envisaged: target identification and validation, discovery of interaction profiles of compounds and targets, detection of potential toxic interactions, repositioning of existing drugs to new therapeutic areas, and many other drug discovery questions [6]. We present three challenging example use cases to demonstrate the requirement for comprehensive integration from multiple data sources to address real world questions. Workflows systems (e.g. KNIME nodes and Pipeline Pilot components) using the Open PHACTS Discovery Platform enable the seamless integration between pathway, target, and compound, permitting retrieval of diverse and complex data from one interface. Additionally, working via the Open PHACTS API solves many unrealized data integration problems for the individual scientist by tackling in the background, data licensing, formatting, and querying issues. Moreover, some of these issues have been further assessed by an empirical evaluation to benchmark improvements across a number of Semantic Web technologies [68]. Most importantly, the platform retains and gives full transparency on data provenance. The Open PHACTS Discovery Platform not only creates connections between heterogeneous data sets but also provides the tools that can help scientist exploit the data available from the API.

The three exemplar use cases demonstrate how the application of Open PHACTS API services can support drug-discovery research. One workflow emphasizes a search strategy across proprietary and public pharmacology databases for a comprehensive identification of chemical compounds targeting the dopamine receptor D_2_. Using a proprietary dictionary generated for in-house data, the different target and compound nomenclatures were reconciled with the public domain data for a comprehensive and meaningful ranking of existing chemical compounds active against the target of interest. The other use case examples leverage the semantically integrated knowledge in the Open PHACTS Discovery Platform on pathways to derive testable hypotheses concerning therapeutic targets. The two pathways, ErbB signaling and Vitamin D metabolism, are representative of a) complex regulatory processes involving a large number of druggable targets and corresponding chemical compounds, and b) comparatively simple and well-defined metabolic processes with few druggable targets. The differences between the two pathways serve to highlight divergent analyses possible via differently combined queries. In one case, pharmacological bioactivity data and its enrichment by integrated annotation terms originating from GO and the ChEBI ontology was turned into a reasonable number of data points, and visualized as heat map representations. While in the other case, key pathway targets (VDR, CYP24A1 and DBP) were explicitly evaluated to identify strategies for designing improved Vitamin D analogues with the desired bioactivity profile.

The workflows developed for the present use cases can be broadly used by drug discovery scientists to exploit the wealth of publicly available information for other targets and pathways of interest. As all the accessed data sets reside in the public domain, the results from the present use cases could, in principle, be derived without the use of the Open PHACTS Discovery Platform. However, it has been previously demonstrated that manual access methods require considerable time and resource investment due to the complexity of data access and licensing for multiple databases, the use of different data formats and identifiers, need for bio- and chemo-informatics expertise and post-processing of data retrieved [1], [6]. Such an exercise is non-trivial for scientists unskilled in programming languages or database management. By providing these example workflows, we hope to encourage the use of the technology to a wide research audience to increases the productivity of both academic and industrial drug discovery projects. Features of the Open PHACTS Discovery Platform useful for our research questions are summarized in Table 6.

**Table 6:** Benefits of using the Open PHACTS Discovery Platform for drug discovery research.

Benefits of using the Open PHACTS platform for drug discovery research
Mapping identifiers to external databases not required
Avoids different interfaces to online knowledge and the need to go back and forth between protein, pathway and bioactivity databases
All integrated data available under “Creative Commons” type licenses
Bioactivity values normalized via QUDT ontology
Getting approved drug status for a list of compounds, including those responsible for off-target or non-approved indications
Getting a list of homologues for a given target
Integration of ontology tags, i.e. from GO and ChEBI and hierarchies with other datasets
Data provenance facilitates enrichment of knowledge from primary literature
Possibility to create specialized API-compatible KNIME nodes to enable other user-defined queries
Existing pipelining workflows can be re-used entirely or in modules to answer other research questions
Results can be easily updated to benefit from future upgrades to the Open PHACTS platform

Together, these examples serve to demonstrate some of the operations made possible via a semantically integrated pharmacology platform. A plethora of other queries requiring the linkage of target-compound-pathway concepts can be envisioned and answered by combining an appropriate sequence of API calls with workflow tools; and, the possibilities for new use cases continue to grow as more data sources are added to the platform. In future releases of the platform, gene-disease association data, protein sequence features, and tissue expression data are scheduled for integration. Additionally, many opportunities exist for the inclusion of new data sets such as text mining data from scientific publications and patents as well as proprietary or commercial data sources [11]. Going forward, the continuation of the infrastructure development and data integration will be carried out in the context of the Open PHACTS Foundation (http://www.openphactsfoundation.org). The Open PHACTS Foundation is the not-for-profit successor organization set up to sustain and continue the growth of the achievements of the Open PHACTS project. Specifically the mission is to maintain a sustainable, open, vibrant and interoperable information infrastructure for applied life science research and development.

## Supporting Information

S1 Fig **Pipeline Pilot workflows for retrieving data for Use Case A; lines 1, 2, and 3 show the components used for retrieving data from Open PHACTS discovery platform; lines 4 and 5 show the components used for retrieving data from Thomson Reuters; and, lines 6, 7, and 8 show the components used for retrieving data from GVKBio GOSTAR.**
(TIF)

S2 Fig **Binary heatmap representation of the pharmacological space in the human ErbB signalling pathway (considering ‘-logActivity values [molar]’ and a cutoff of 6); abscissae: targets with ChEMBL target ID's; ordinate: compounds; red bars indicate ‘actives’, blue bars ‘inactives’, grey areas indicate that no activity value was reported.**
(TIF)

S3 Fig **Binary heatmap representation for compounds annotated with ‘antineoplastic agent’ in ChEBI (considering ‘-logActivity values [molar]’ and a cutoff of 6); abscissae: targets with ChEMBL target ID's; ordinate: compounds; red bars indicate ‘actives’, blue bars ‘inactives’, grey areas indicate that no activity value was reported.**
(TIF)

S1 Table **List of current resources available through the Open PHACTS Discovery Platform.**
(XLSX)

S2 Table **Examples of free text and URI inputs used in the API calls.**
(XLSX)

S3 Table **List of all GO ‘biological process’ terms that have been annotated to at least 5 of the 23 prioritized targets (plus ChEMBL target IDs of those targets).**
(XLSX)

S4 Table **List of all ChEBI classification terms for the 23 prioritized targets that have been annotated to at least 6 compounds.**
(XLSX)

S5 Table **Specificity of compounds targeting proteins in the Vitamin D pathway.**
(XLSX)

S6 Table **Additional pathways for targets in the Vitamin D pathway.**
(XLSX)

S7 Table **List of VDR and DBP orthologues and corresponding bioactivity records.**
(XLSX)

S1 File **Organic molecules active against DRD2 retrieved from Open PHACTS API.**
(XLSX)

S2 File **Pharmacological profile of compounds with ChEBI term ‘antineoplastic agent’.**
(XLSX)

S3 File **All compound bioactivity data for targets in the Vitamin D pathway.**
(XLS)

S4 File **Compounds tested against DBP and VDR orthologues.** KNIME workflows: in http://www.myexperiment.org/groups/1125.html. Pipeline Pilot script: in https://community.accelrys.com/docs/DOC-6473.
(XLS)

S1 Method **Selection of pathway use cases.**
(DOCX)
