# Supplementary figures and images for: The Application of the Open Pharmacological Concepts Triple Store (Open PHACTS) to Support Drug Discovery Research
Source: PLoS One. 2014 Dec 18;9(12):e115460. doi: 10.1371/journal.pone.0115460 (PMC4270790; doi:10.1371/journal.pone.0115460)

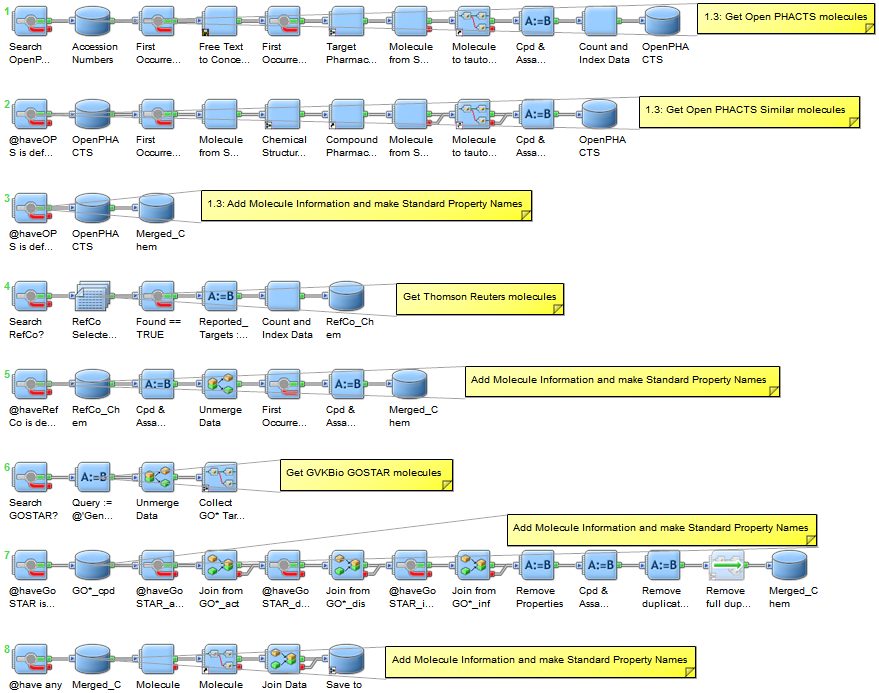

Supplement: S1 Fig — Pipeline Pilot workflows for retrieving data for Use Case A; lines 1, 2, and 3 show the components used for retrieving data from Open PHACTS discovery platform; lines 4 and 5 show the components used for retrieving data from Thomson Reuters; and, lines 6, 7, and 8 show the components used for retrieving data from GVKBio GOSTAR. (TIF) [file pone.0115460.s001.tif]

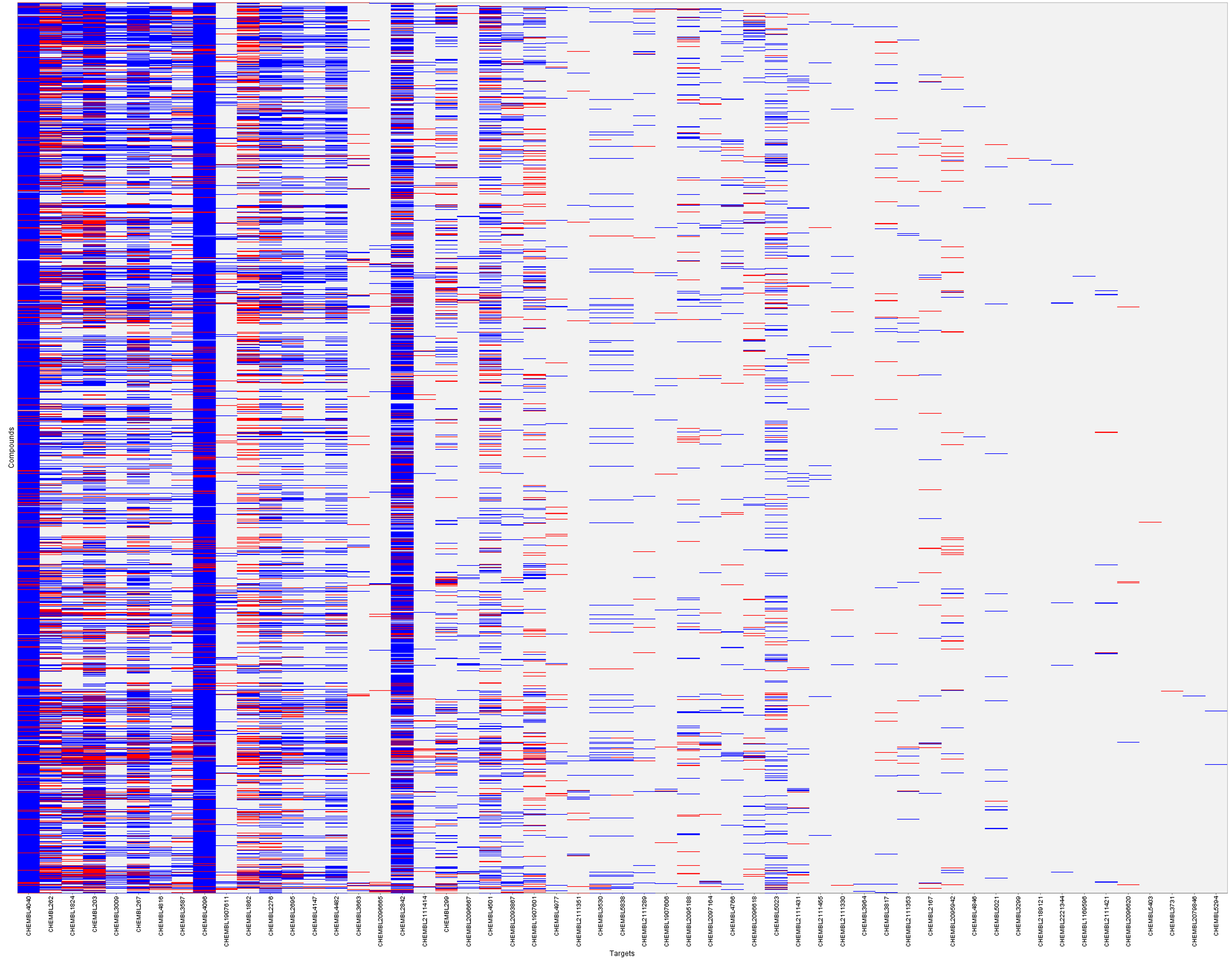

Supplement: S2 Fig — Binary heatmap representation of the pharmacological space in the human ErbB signalling pathway (considering ‘-logActivity values [molar]’ and a cutoff of 6); abscissae: targets with ChEMBL target ID's; ordinate: compounds; red bars indicate ‘actives’, blue bars ‘inactives’, grey areas indicate that no activity value was reported. (TIF) [file pone.0115460.s002.tif]

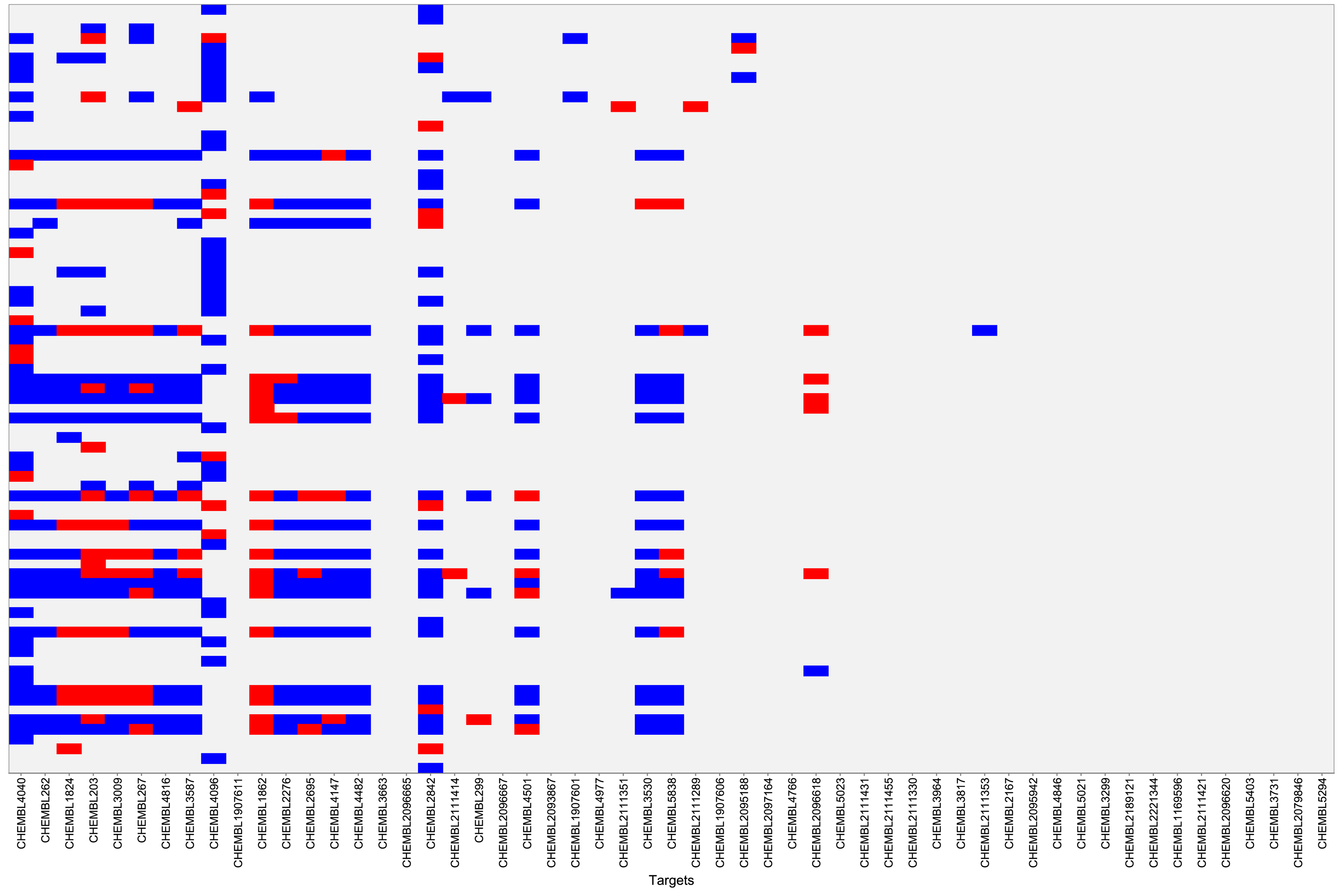

Supplement: S3 Fig — Binary heatmap representation for compounds annotated with ‘antineoplastic agent’ in ChEBI (considering ‘-logActivity values [molar]’ and a cutoff of 6); abscissae: targets with ChEMBL target ID's; ordinate: compounds; red bars indicate ‘actives’, blue bars ‘inactives’, grey areas indicate that no activity value was reported. (TIF) [file pone.0115460.s003.tif]
