# Supplementary material for: The Application of the Open Pharmacological Concepts Triple Store (Open PHACTS) to Support Drug Discovery Research
Source: PLoS One. 2014 Dec 18;9(12):e115460. doi: 10.1371/journal.pone.0115460 (PMC4270790; doi:10.1371/journal.pone.0115460)
Supplement: S1 Method — Selection of pathway use cases. (DOCX) [file pone.0115460.s015.docx]

**SUPPLEMENTARY METHODS**

**Method S1-Selection of pathway use cases:**

Consistent with the idea of broad applicability, we chose to build our use-cases on well curated and relevant pathways selected as described below:

1. List of 20 ‘most viewed’ human pathways from Wikipathways (31^st^ May 2013)
2. Quality of pathway (curation tags in Wikipathways, minimum annotation errors)
3. Linkage to disease ontology terms
4. Number of PMIDs associated with each pathway
5. Number of compounds and known drugs hitting every protein in a given pathway adding a filter for endpoints 'IC_50_', 'Ki', 'Activity', 'Potency', 'Kd', 'Inhibition', and 'EC_50_’
